# Suppressive Role of Lactoferrin in Overweight-Related Female Fertility Problems

**DOI:** 10.3390/nu14050938

**Published:** 2022-02-22

**Authors:** Ban Sato, Seiya Kanai, Daiki Sakaguchi, Kodai Yajima, Yu Matsumoto, Kazunori Morohoshi, Shinji Kagaya, Nobuo Izumo, Minoru Ichinose, Woojin Kang, Mami Miyado, Kenji Miyado, Natsuko Kawano

**Affiliations:** 1 Laboratory of Regulatory Biology, Department of Life Sciences, School of Agriculture, Meiji University, 1-1-1 Higashimita, Tama, Kawasaki 214-8571, Japan; bansato@meiji.ac.jp (B.S.); nekomaru0@gmail.com (S.K.); b01133040d@edu.teu.ac.jp (D.S.); cf200417@meiji.ac.jp (K.Y.); cf210430@meiji.ac.jp (Y.M.); k_morohoshi@meiji.ac.jp (K.M.); 2 NRL Pharma, Inc., East Block 203, Kanagawa Science Park, 3-2-1 Sakado, Takatsu-Ku, Kawasaki 213-0012, Japan; skagayas@gmail.com; 3 Laboratory of Pharmacotherapy, Yokohama University of Pharmacy, 601 Matano, Totsuka, Yokohama 245-0066, Japan; n.izumo@hamayaku.ac.jp; 4 Department of Reproductive Biology, National Research Institute for Child Health and Development, 2-10-1 Okura, Setagaya, Tokyo 157-8535, Japan; ichinose-m@ncchd.go.jp (M.I.); kwjbear@gmail.com (W.K.); 5 Department of Molecular Endocrinology, National Research Institute for Child Health and Development, 2-10-1 Okura, Setagaya, Tokyo 157-8535, Japan; miyado-m@ncchd.go.jp

**Keywords:** lactoferrin, overweight, obesity, female fertility problems

## Abstract

The secretory glycoprotein lactoferrin (LF) is suggested to ameliorate overweight regardless of non-genetic or genetic mechanisms. Although maternal overweight represents a key predictor of offspring growth, the efficacy of LF on fertility problems in overweight and obese mothers remains unknown. To address this issue, we examined the effect of LF ingestion by analyzing overweight mice (Institute of Cancer Research (ICR) mice with high-fat diets; HF mice) and obese mice (*leptin*-deficient mice with type II diabetes; *ob/ob* mice). Plasma insulin, leptin, glucose, and cholesterol levels were measured, and thermal imaging and histological analysis were employed. The litter size of HF females was reduced due to miscarriage, which was reversed by LF ingestion. In addition, LF ingestion suppressed overweight prevalence in their offspring. The component analysis of the maternal blood demonstrated that glucose concentration in both HF females and their offspring was normalized by LF ingestion, which further standardized the concentration of insulin, but not leptin. LF ingestion was unable to reverse female infertility in *ob/ob* mice, although their obesity and uterine function were partially improved. Our results indicate that LF upregulates female fertility by reinforcing ovarian and uterine functions in females that are overweight due to caloric surplus.

## 1. Introduction

Being overweight, defined as a non-genetic event with excess fat accumulation throughout the body, increases the risk of health problems such as high blood pressure, heart disease, dyslipidemia, and diabetes [1]. High-fat (HF) diets induce overweight and, subsequently, diabetes in rodents, including rats and mice [2]. The animals share common symptoms with overweight humans, such as increased fat mass, hyperinsulinemia, and hyperglycemia [2]. Similarly, obesity originating from genetically inherited genomic mutations, and often displaying circadian rhythm disruption, exhibits excessive fat accumulation [1]. Homozygous mutation of the *ob* gene leads to a defect in leptin production, resulting in insulin-resistant obesity in mice (*ob/ob* mice) [3]. Regardless of overweight or obesity, there are considerable concerns for pregnant women due to the correlation with adverse pregnancy outcomes, such as caesarean section complications, as well as mother and offspring health problems [4]. Quaresima et al. (2021) enlightened women on the risk of gestational diabetes mellitus (GDM) [5]. Weight management during pregnancy is necessary for non-problematic reproduction despite increased appetite and reduced exercise.

**Figure 1:**
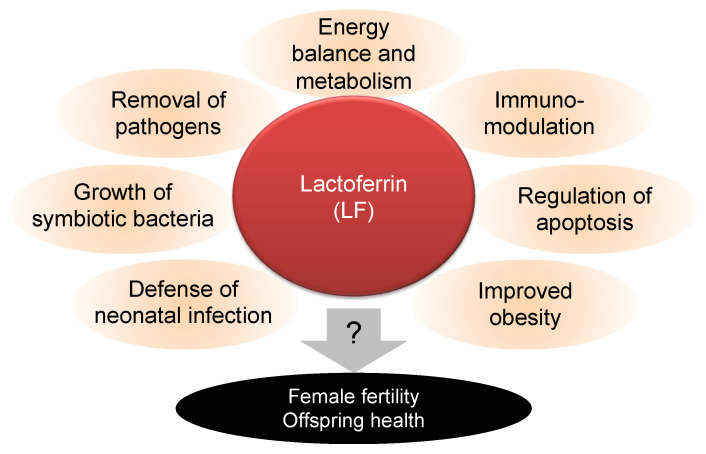
Beneficial effects of lactoferrin (LF) on health problems. LF is a multifunctional protein that contributes to various physical events, including the suppression of neonatal infection, growth of symbiotic bacteria (and, conversely, the suppression of pathogenic bacteria), suppression of obesity closely linked to energy balance and metabolism, immunomodulation, and regulation of apoptosis. Otherwise, its efficacy on female fertility and offspring health is unknown.

Lactoferrin (LF) is an iron-binding multifunctional protein detected on mucosal surfaces of epithelial and immune cells [6] (Figure 1). LF is abundant in maternal milk and detected in lower amounts in exocrine secretions, including saliva, tears, semen, and vaginal and gastrointestinal fluids [7]. LF is considered a growth promoter for Bifidobacterium, a bacterium that predominantly inhabits the human intestines [8]. LF ingestion contributes to the clinical outcomes of various diseases by enhancing the host defense through inflammatory responses [7]. LF contained in maternal milk exhibits protective effects against neonatal infections, particularly in neonates with low birth weight [6]. LF uptake reduces body weight in human and animal models of obesity [9]. LF is also detected in human oviductal secretions and binds to sperm, as well as eggs, thus potentially modulating fertilization [10]. However, its relationship with reproductive ability and neonatal health in overweight and obese women is not well understood. Therefore, there is an urgent need to discover effective and safe drugs, as well as functional foods, to aid the pregnancy and birth processes in overweight women. Researchers have collected evidence supporting the effectiveness of maternal milk-derived proteins such as LF against metabolic diseases, including overweight and obesity. Correspondingly, LF contributes to the energy balance and metabolism directly or indirectly [11].

During pregnancy, although caloric intake increases, physical activity decreases in most women, leading to overweight. Offspring of overweight or obese women are at increased risk of being born large for gestational age and become overweight, frequently leading to obesity in childhood and even during adulthood [12]. Correspondingly, maternal body weight increases before and during pregnancy, which is a major determinant of metabolic outcomes leading to cardiovascular diseases in offspring [13]. However, the effectiveness of LF on fertility problems, including anovulatory infertility and preterm birth in overweight and obese mothers, remains unknown.

In the present study, we investigated the positive effects of LF ingestion on the reproductive ability of HF diet-induced obese mice (HF mice) and *ob/ob* female mice, as well as the health of their neonates.

## 2. Materials and Methods

### 2.1. Diet

Normal (CE2) and HF diets (HDF32) were purchased from CLEA Japan, Inc. (Tokyo, Japan). Bovine LF was purchased from Taura-Bio (Lactoferrin Powder 500201; Tokyo, Japan).

### 2.2. Animals

Female (4 weeks old) and male (3–5 months old) ICR mice were purchased from Japan SLC Inc. (Shizuoka, Japan.) Female *ob/ob* (C57BL6JHamSlc-ob/ob, 5 weeks old) and male *ob/ob* (C57BL6JHamSlc-ob/ob, 10 weeks old) mice were purchased from Japan SLC Inc. (Shizuoka, Japan). Mice were housed under standardized light-dark cycle conditions (lights on at 7:00 a.m., off at 7:00 p.m.). Food and water were provided *ad libitum*. 

### 2.3. Breeding with HF Diets and LF Ingestion

ICR female mice were fed with normal and HF diets after 4 weeks of age and mated with ICR male mice. Female mice were divided into two groups: normal water or water containing 8% (*w*/*v*) LF. We used water without any proteins, such as BSA and casein, because protein-rich diets have no influence on blood glucose [14].

### 2.4. Measurement of Body Weight

Five female mice, belonging to each of the two or four groups (mice fed with normal and HF diets, and mice provided with normal and LF-containing water), were weighed between 10:00 a.m. and 12:00 p.m. from 4 weeks of age across pregnancy and delivery. Concomitantly, the body weight of the offspring was measured from the day after birth (0 days) and compared with that of their mothers.

### 2.5. Estimation of Female Fertility (Litter Size and Implantation)

To determine the litter size, the number of pups delivered by ICR and *ob/ob* female mice was recorded by placing their counterparts in the cage. After delivery, the number of implantation marks in the uterus was counted as implanted embryos. The difference between implanted embryos and actual litter size indicates the number of miscarried fetuses.

### 2.6. Quantitative Estimation of Plasma Key Substances

To determine the plasma key substance concentrations, blood samples were collected from the mouse orbital sinus with heparinized calibrated pipets (Drummond Scientific Company, Broomall, PA, USA), and the plasma was prepared by centrifugation at 1000× *g* for 10 min. Plasma insulin levels were measured using a Morinaga Ultra Sensitive Mouse/Rat Insulin ELISA kit (Morinaga Bioscience Laboratory, Yokohama, Japan). Plasma leptin levels were measured using a Morinaga Mouse/Rat Leptin ELISA kit (Morinaga Bioscience Laboratory). Plasma glucose levels were measured using an animal glucometer (LAB Gluco; ForaCare Japan Co., Ltd., Tokyo, Japan). Plasma cholesterol levels were measured with a cholesterol E-test (Wako Pure Chemical Industries, Osaka, Japan).

### 2.7. Thermal Image by Infrared (IR) Thermography

To measure the surface temperature of the mouse, we used an HT-18 thermal imaging camera (Dongguan Xintai Instrument Co., Ltd., Dongguan, China) with a temperature range from −20 °C to 300 °C and a thermal sensitivity less than 0.07 °C. Temperature measurements were conducted under controlled temperature (23 °C) conditions.

### 2.8. Histological Analysis

A histochemical method was used to observe the uterine tissues, as described previously [15]. Briefly, after *ob/ob* female mice were killed at 11 weeks of age, their uteri were isolated. Next, 5 µm sections were prepared from paraffin-embedded tissues. After deparaffinization, the sections were stained with hematoxylin and eosin.

### 2.9. Statistical Analysis

Significant differences were calculated using Student’s *t*-test, and *p*-values < 0.05 were considered statistically significant. Results are expressed as mean ± standard error.

## 3. Results

### 3.1. Effect of LF on Overweight Female Mice before and after Pregnancy

**Figure 2:**
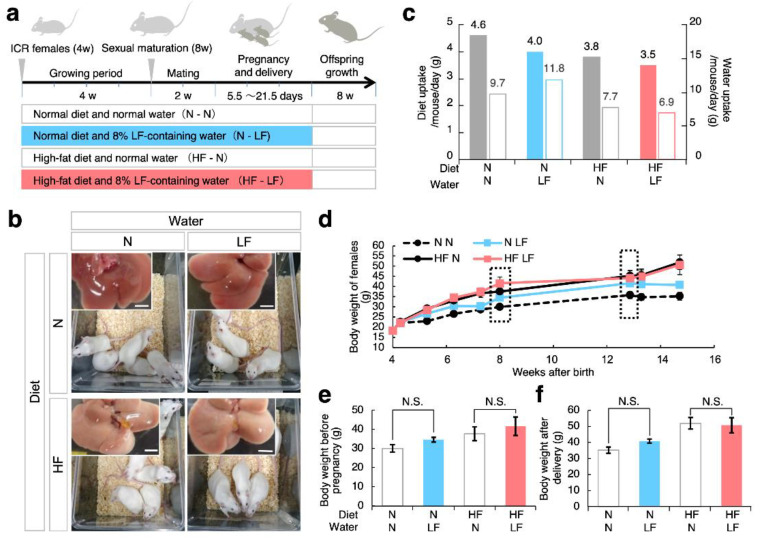
LF ingestion in overweight female mice. (**a**) Experimental flow. Overweight was induced by feeding Institute of Cancer Research (ICR) female mice with a high-fat (HF) diet from 4 weeks of age to weaning. A normal diet (N) was used as well. In parallel, LF-containing or LF-free water was provided to mice. After weaning, the offspring were fed a normal diet and LF-free water. (**b**) Breeding environment and gross views of livers. Scale bars: 5 mm. (**c**) Effect of LF ingestion on diet and water uptakes in high-fat diet-induced obese mice (HF mice). Solid bars represent diet uptake in HF female mice with LF ingestion (each group: n = 5). Open bars represent water uptake in HF female mice with LF ingestion (each group: n = 5) (N-N: normal diets and water; N-LF: normal diets and water containing LF; HF-N: HF diets and water; HF-LF: HF diets and water containing LF). (**d**) Body weight of females in each group (n = 5). (**e**) Body weight of females at 8 weeks of age. (**f**) Body weight of females at 15 weeks of age after delivery. Values are expressed as means ± standard error (SE).

Inbred strains of mice, such as C57BL/6, CBA, and BALB/c, are more widely used than outbred strains of mice, such as ICR, because phenotypic variations are larger in outbred strains than in inbred strains. In the present study, we utilized ICR mice because we aimed to identify dietary effects picked up in their genetic variations. As depicted in the experimental flow (Figure 2a), the female mice were divided into two groups: mice fed HF diets (HF mice) and normal diets. Furthermore, these groups were separated into two other groups: mice provided with water containing 8% LF and normal water. The *ad libitum* administration of HF diets and LF to female mice was continued from 4 weeks of age throughout the pregnancy, delivery, and weaning.

As shown in Figure 2b, five mice were bred in the same cage, and adipose tissues and liver were accumulated in HF female mice, regardless of the presence or absence of LF in the drinking water. The daily water uptake and diet consumption measurements (Figure 2c) indicated that LF ingestion tended to reduce the diet intake, regardless of diet type.

To examine the effect of LF ingestion on maternal overweight, we serially measured the body weights of female mice (d). In HF females, maternal weight tended to be higher than that in mice fed normal diets. However, LF ingestion did not seem to affect this trend. To address this point, we selected two measurement stages: before and after pregnancy (Figure 2e,f). As expected, LF ingestion did not seem to affect maternal overweight induced by the HF diets.

### 3.2. Effect of LF on Fertility in HF Females and Their Offspring Weight

**Figure 3:**
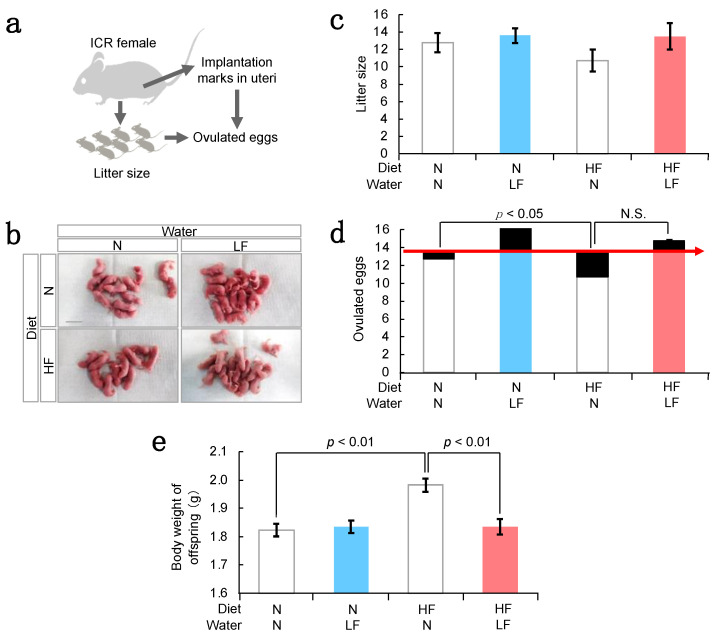
Effect of LF ingestion on ovarian function and offspring weight. (**a**) Experimental flow. As shown in Figure 2a, a HF diet and LF were administered to ICR female mice. The litter size and implantation marks observed in the postpartum uterus were counted. Difference between litter sizes and the number of total ovulated eggs corresponded to the number of embryos lost at implantation. (**b**) Image of offspring. Scale bars: 2 cm. (**c**) Litter size from females in each of groups (N-N; Normal diets and water: n = 4; N-LF: Normal diets and water containing LF: n = 5; HF-N: HF diets and water: n = 4; HF-LF: HF diets and water containing LF: n = 4). Values are expressed as means ± SE. (**d**) The number of implanted embryos from the mothers in each group (N-N: n = 4; N-LF: n = 5; HF-N: n = 4; HF-LF: n = 4). Embryos lost was indicated as a black box. The value for control mice was expressed as a red arrow. (**e**) Body weight of offspring (0 day postpartum) (N-N: n = 51; N-LF: n = 68; HF-N: n = 43; HF-LF: n = 54). Values are expressed as means ± SE.

To investigate the effect of LF ingestion on the maternal reproductive ability, we counted the number of offspring (litter size) and the number of histological signs of embryos implanted but not developed to term (implantation marks) (Figure 3a). The number of implantation marks was estimated using the ovulated eggs from ovaries and the difference between implantation marks and litter size means the miscarriage. The litter size tended to be reduced in HF females, and LF ingestion reversed this trend (Figure 3b,c). Similarly, the number of ovulated eggs was comparable between mice fed with high-fat and normal diets. However, the number of implantation marks was higher in HF mice (Figure 3d). In contrast, LF ingestion reduced the number of implantation marks and increased the number of ovulated eggs, subsequently increasing the litter size, regardless of the presence or absence of HF diet. Based on this, we hypothesize that LF has the potential to maintain ovarian function and stimulate the ovulation of eggs compatible with the developmental ability.

In general, when female weight increases during pregnancy, offspring weight is elevated synchronously [12]. We measured the body weights of HF female offspring (Figure 3e) and observed that the HF diet increased the offspring weight—an effect that was reversed by LF ingestion. Thus, we derived that LF ingestion could improve uterine or placental function, as well as ovarian function, subsequently suppressing offspring overweight.

### 3.3. Serum Components in HF Females and Their Offspring

**Figure 4:**
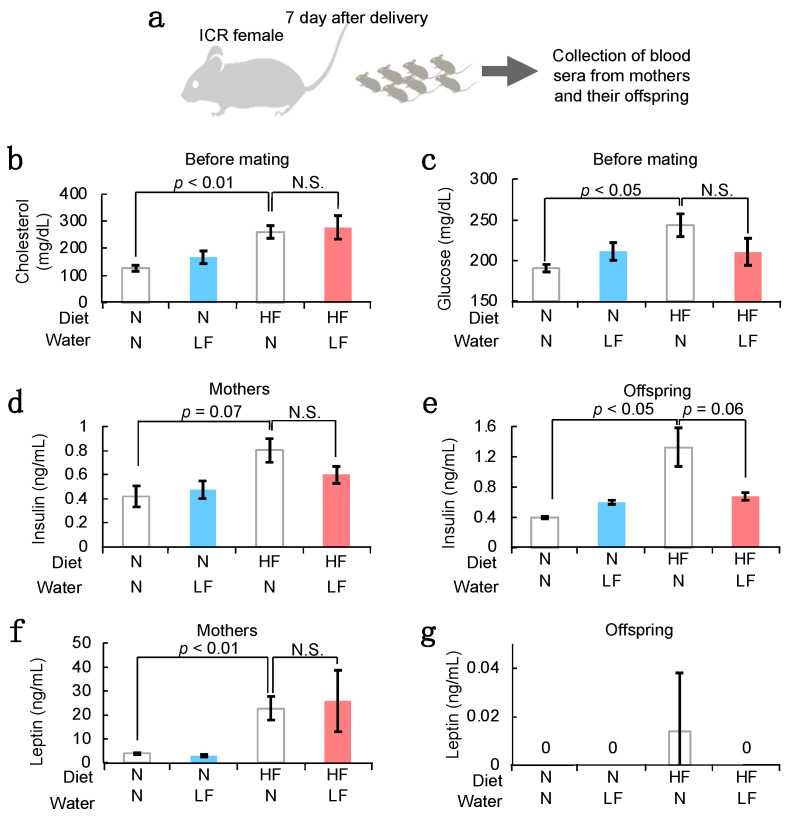
Measurement of serum components in HF females and their offspring. (**a**) Experimental flow. As depicted in Figure 2a, a HF diet and LF were administered to ICR female mice. Serum samples were collected from the orbital sinus of anesthetized ICR females and the carotid artery of their offspring. (**b**) Serum cholesterol concentration in female mice (8 weeks old) (n = 5). (**c**) Glucose concentration in female mice (8 weeks old) (n = 5). (**d**) Serum insulin concentration in female mice 1 week after delivery (each group: n = 3). (**e**) Serum insulin concentration in the offspring (7 days postpartum) (each group: n = 3). (**f**) Serum leptin concentration in female mice (1 week after delivery) (each group: n = 3). (**g**) Serum leptin concentration in the offspring (7 days postpartum) (each group: n = 3). Values are expressed as means ± SE.

To investigate the effect of LF ingestion on serum characteristics, we measured the serum concentrations of overweight-related factors (cholesterol, glucose, leptin, and insulin) (Figure 4a). Cholesterol levels increased in overweight mothers, and LF ingestion did not affect its serum concentration (Figure 4b). Glucose levels also increased in HF mothers. However, LF ingestion tended to reduce its serum concentration (Figure 4c). Correspondingly, insulin levels increased in the HF mothers and their offspring 7 days after birth, and LF ingestion tended to reduce the insulin concentration (Figure 4d,e). In contrast, leptin levels increased in the HF mothers, and LF ingestion did not affect the leptin concentration in both mothers and their offspring (Figure 4f,g).

### 3.4. Effect of LF Ingestion on Fertility Problems in ob/ob Females

**Figure 5:**
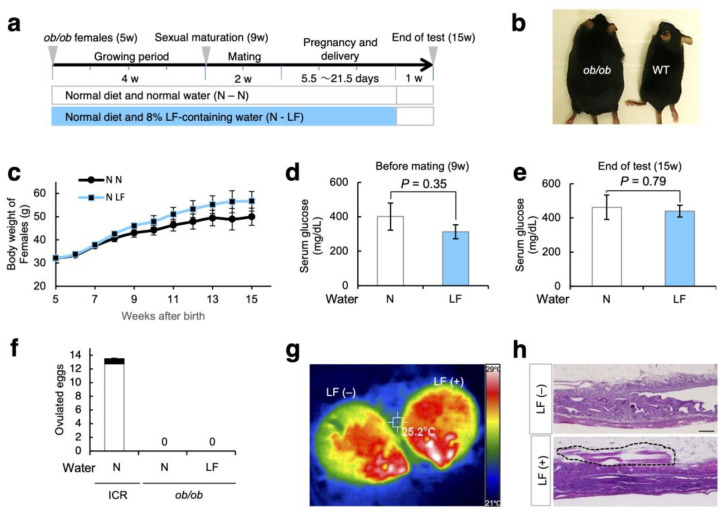
Effect of LF ingestion in *ob/ob* female mice. (**a**) Experimental flow. As depicted in Figure 2a, a HF diet and LF were administered to *ob/ob* female mice. (**b**) Appearance of 7-week-old *ob/ob* and wild-type female (WT) (C57BL6JHamSlc) mice without treatment. (**c**) Body weight of *ob/ob* female mice from the initiation of the HF diet and LF supplementation (each group: n = 5). Values are expressed as means ± SE. (**d**) Serum glucose concentration of *ob/ob* females (9 weeks old) (each group: n = 5). Values are expressed as means ± SE. (**e**) Serum glucose concentration of *ob/ob* females at the end of test (each group: n = 5). Values are expressed as means ± SE. (**f**) Number of ovulated eggs from mother in each group (ICR-N: n = 4; *ob/ob*-N: n = 5; *ob/ob*-LF: n = 5). Difference in litter size and the number of total ovulated eggs corresponded to the lost number of embryos at implantation and are indicated by the black box. (**g**) Thermography. (**h**) Histological examination of the uterus. The regions surrounded by a dotted line indicate muscular tissues. Scale bars: 200 µm.

To explore the medicative effect of LF on obesity-related fertility problems, we examined the *ob/ob* mice lacking *leptin* genes. As depicted in Figure 5a, *ob/ob* females were divided into two groups: mice provided with LF-containing water and normal water. The administration of LF to female mice was continuous from 5 to 14 weeks of age.

As shown in Figure 5b, the *ob/ob* females (7 weeks old) were larger than the WT females. This tendency was comparable to that shown in Figure 2c, indicating that LF ingestion had no effect on the body weight of *ob/ob* mothers (Figure 5c). Moreover, the concentration of serum glucose was unchanged in *ob/ob* females before mating and after the tests, regardless of the presence or absence of LF in the drinking water (Figure 5d,e).

As reported previously [16], female *ob/ob* mice are infertile, although there are small chances of reproduction with young male mice. The *ob/ob* females mated with their male counterparts at a similar frequency, regardless of the presence or absence of LF ingestion (Supplementary Figure S1a). However, the *ob/ob* females were still infertile even after LF ingestion (Figure 5f). To examine the effect of LF on the metabolism of *ob/ob* females, we measured the surface body temperature before and after intraperitoneal LF injection using thermography. Subsequently, the surface body temperature was higher in *ob/ob* mice after LF injection than before LF injection (Figure 5g; Supplementary Figure S1b). Although the morphology of the ovary did not change, the disrupted uterine muscles were rebuilt in the space between the uterine tissues and adipose tissues in LF-injection *ob/ob* females (Figure 5h and Supplementary Figure S2).

## 4. Discussion

In the present study, we demonstrated that LF ingestion can improve fertility in overweight/obese females and suppress the health problems of their offspring. Our results showed the following: (1) HF diet-fed female mice became overweight and LF ingestion had no significant effect on their body weight, whereas the weight of their offspring was reduced to normal levels; (2) LF ingestion increased the number of ovulated eggs, indicating enhancement of ovarian function; (3) LF ingestion had no impact on the infertility of *ob/ob* females, whereas LF injection improved muscular formation in the uterus. LF-containing diets have been already shown to have multiple health benefits (Figure 1). In addition, our results suggest that LF ingestion by mothers improves offspring health, indicating that LF eliminates the negative spiral from overweight females to offspring.

### 4.1. Possible Mechanisms of LF for Preventing Fetal Macrosomia

As reported previously [17], recombinant LF proteins prevent preterm delivery. Since LF and lipopolysaccharide (LPS) were injected into the vagina of pregnant mice, LF might exhibit anti-inflammatory and/or antibacterial activity inside the female reproductive tract. Correspondingly, our results provide the first evidence that LF is a promising pharmacological agent for fetal health, especially with macrosomia (Figure 3c,e).

In humans, fetal macrosomia, defined as a birth weight ≥ 4000 g, affects 12% of newborns from healthy women and 15–45% of newborns from women with GDM [18]. In GDM patients, an excess amount of blood glucose passes through the placenta into the fetal circulation, causing macrosomia and truncal fat deposition. The high fetal demand for glucose requires the presence of a rapid, high-volume system for maternal-fetal transfer mediated by placental glucose transfer (GLUT) because of the absence of significant gluconeogenesis in the fetus [19]. Moreover, in mice, tumor necrosis factor-α (TNF-α) and insulin-like growth factor 1 (IGF1) promote the expression of glucose transporter protein type 1 (GLUT-1), causing hyperglycemia in the placenta, which accelerates the overgrowth of the fetus [20,21]. LF ingestion leads to LF transport from the intestinal lumen into the blood circulation [22], resulting in LF arriving at the placenta. Intravenous injection of LF in vivo was shown to decrease the release of LPS-induced TNF-α [23]. Moreover, intragastric intervention with LF increased IGF1 mRNA expression and promoted bone formation in a senile osteoporosis mouse model [24]. This evidence suggests that LF ingestion might regulate GLUT-1 activity in the placenta due to changes in the expression of TNF-α and IGF1, which may suppress the transfer of excess glucose from the mother to the fetus. Correspondingly, we showed that ingestion of LF by the mother normalized the insulin level in the offspring (Figure 4e).

### 4.2. LF Upregulates the Ovarian Function

Miscarriages are underlying by multiple acquired factors, including lipotoxicity and hyperglycemia. In general, overweight mice display lipotoxicity, such as lipid accumulation in oocytes and the supporting cells of oocytes, cumulus, and granulosa cells, leading to endoplasmic reticulum stress, mitochondrial dysfunction, and ultimately apoptosis [25,26]. Hyperglycemia in the early stages of pregnancy is associated with inadequate oocyte maturation and blastocyst development, which increases the incidence of implantation failure [27]. Here, we showed that a HF diet increased the miscarriage incidence in mice (Figure 3d). Furthermore, previous studies have shown that there is a relationship between early spontaneous miscarriages and pregestational diabetes in women with poor glycemic control [28,29].

Here, we showed that LF ingestion increased the total number of eggs ovulated in HF females (Figure 3d). Concomitantly, LF ingestion improved blood glucose concentration and did not affect the serum cholesterol levels in mice (Figure 4b), assuming that ovary function could be enhanced by controlling the blood glucose concentration. One of the ovulation disorders, polycystic ovary syndrome (PCOS), is caused by hyperandrogenism with insulin resistance in women with hyperglycemia [30]. LF ingestion might improve PCOS-like symptoms by controlling the blood glucose concentration. PCOS is a common and heterogeneous endocrine disorder in women of reproductive age, with several relevant clinical implications [31]. The improvement of PCOS by LF might be of great social importance. However, to support these claims, further studies are necessary.

In addition to the effects of LF on female mice fed HF diets, LF ingestion increased the total number of ovulated eggs from females with normal diets (Figure 3d), implying that LF elevates ovary function even in healthy conditions. Ovulation is generally controlled by endocrine factors, inflammation, and apoptotic signals [32,33]. Apoptosis is essential for the selection of follicles available during ovulation and the control of the number of ovulated eggs [34]. In a previous report [35], the injection of an excess amount of TNF-α into the bursa increased the number of apoptotic cells and reduced the number of ovulated eggs. This evidence suggests that the optimum amount of apoptosis factors is required for the success of ovulation. Thus, LF ingestion may adjust the expression of apoptotic factors such as TNF-α during ovulation with maximum efficiency.

However, LF was unable to improve ovarian function in *ob/ob* females (Figure 5f). Leptin-deficient mice exhibit reduced gonadotropin-releasing hormone (GnRH) secretion, leading to gonadotropin deficiencies and hypogonadism [36]. Leptin itself induces GnRH secretion and elicits luteinizing hormone (LH)-independent ovulation [37]. Since leptin functions in various aspects of metabolism and reproduction, LF ingestion may be incapable of compensating leptin deficiency in females, even though the surface temperature and the appearance of the uterus are seemingly improved (Figure 5g,h).

**Figure 6:**
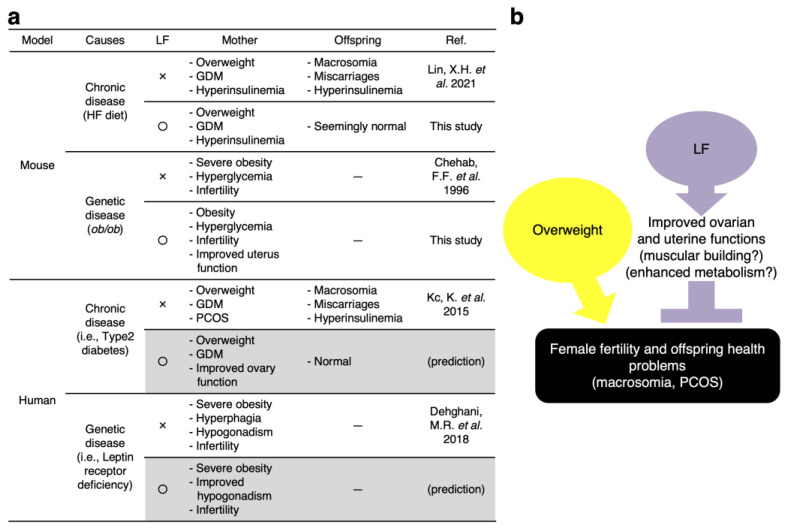
(**a**) Summary of our findings and corresponding previous works [16,18,41,42]. (**b**) The possible function of LF as a reproductive medicine. LF suppresses female fertility problems by maintaining ovarian and uterine functions. Subsequently, LF might eliminate the negative disease proclivities that non-pathogenic overweight and pathogenically obese females pass to their progeny.

Accumulating evidence indicates that maternal health problems represent a major determinant of health problems in offspring throughout childhood and adulthood [38]. For example, if overweight women resolve their weight problems prior to their pregnancy, the offspring have a reduced risk of overweight and obesity [39,40]. Even though weight control is difficult following pregnancy, our results indicate that LF glycemic control recovers fetal metabolism and the reproductive activity of the mother (Figure 6). Thus, we suggest that LF might represent a remedy against the passing over of disease proclivities from non-pathogenic overweight mothers to their offspring.

## 5. Patents

This paper contains a part of the contents of the Japanese patent publication, 2019-034924A, “The mother during pregnancy health improving agent.” published on 7 March 2019.

## Supplementary Materials

The following supporting information can be downloaded at: https://www.mdpi.com/article/10.3390/nu14050938/s1. Supplementary Figure S1: Effect of LF ingestion on *ob/ob* female mice. Supplementary Figure S2: Histological analysis of uterine tissues in *ob/ob* mice.
