# Supplementary material for: Suppressive Role of Lactoferrin in Overweight-Related Female Fertility Problems
Source: Nutrients. 2022 Feb 22;14(5):938. doi: 10.3390/nu14050938 (PMC8912823; doi:10.3390/nu14050938)
Supplement: Supplementary file 1 [file nutrients-14-00938-s001.zip › supplementary Figure S1.pdf]

Supplementary Figure S1

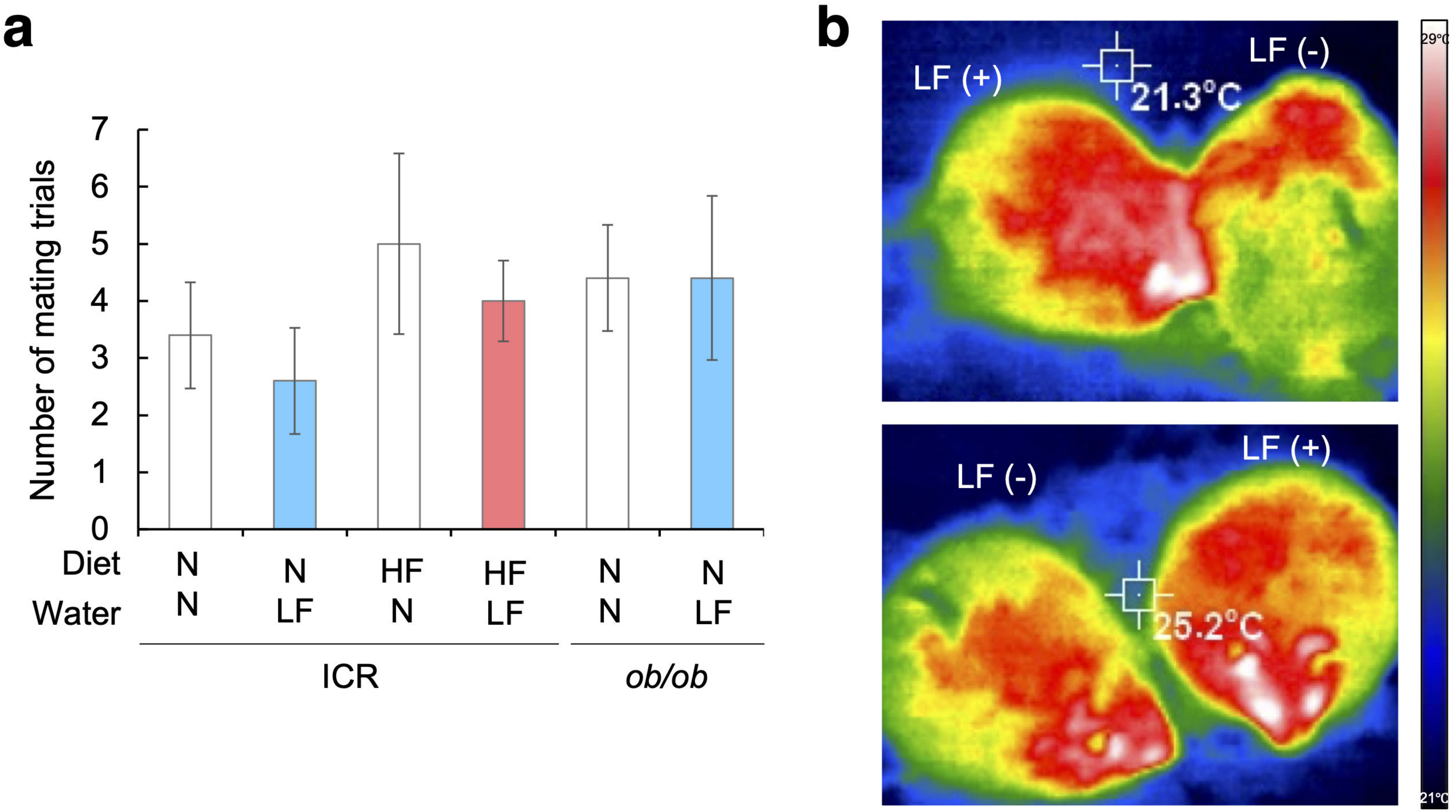

Supplementary Figure S1. Effect of LF ingestion in *ob/ob* female mice. (**a**) The number of mating trials until mating plug was observed (each group: n = 5). (N-N: Normal diets and water; N-LF: Normal diets and water containing LF; HF-N: HF diets and water; HF-LF: HF diets and water containing LF). (**b**) Thermography
