# Supplementary material for: Suppressive Role of Lactoferrin in Overweight-Related Female Fertility Problems
Source: Nutrients. 2022 Feb 22;14(5):938. doi: 10.3390/nu14050938 (PMC8912823; doi:10.3390/nu14050938)
Supplement: Supplementary file 1 [file nutrients-14-00938-s001.zip › supplementary Figure S2.pdf]

Supplementary Figure S2

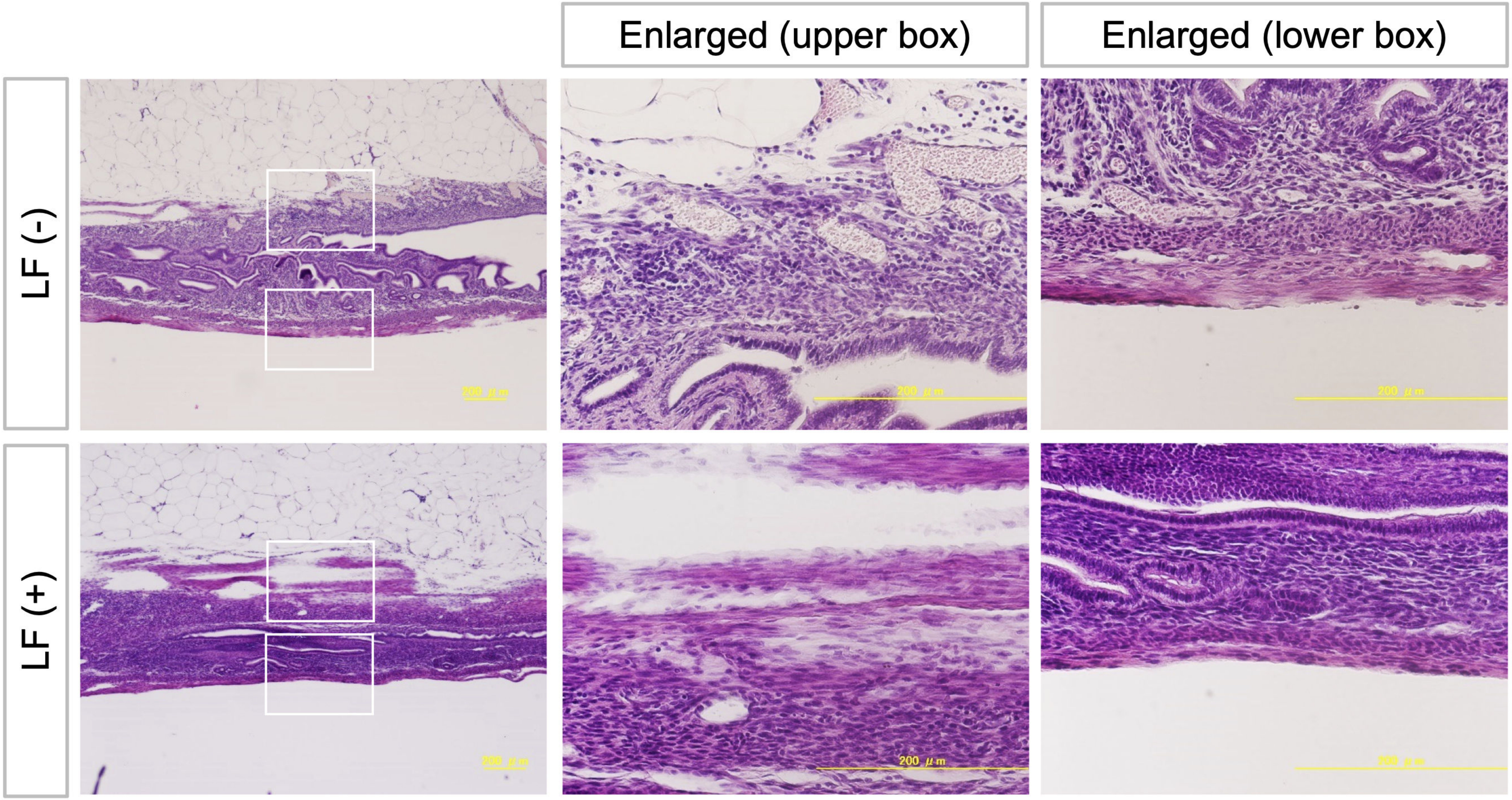

Supplementary Figure S2. Histological analysis of uterine tissues in *ob/ob* mice. Boxed regions were enlarged in the middle and right panels. Scale bars, 200 µm.
